# The difference in red blood cell distribution width from before to after thrombolysis as a prognostic factor in acute ischemic stroke patients: A 2-year follow-up

**DOI:** 10.3389/fneur.2022.1011946

**Published:** 2022-10-13

**Authors:** Yanyan Jiang, Chuancheng Ren, Aydos Alimujiang, Yuncheng Wu, Dongya Huang, Weiting Yang

**Affiliations:** ^1^Department of Neurology, Shanghai East Hospital, Tongji University School of Medicine, Shanghai, China; ^2^Department of Neurology, Shanghai General Hospital, Shanghai Jiao Tong University School of Medicine, Shanghai, China

**Keywords:** acute ischemic stroke, red blood cell distribution width, recombinant tissue plasminogen activator, predictive factor, 2-year follow-up

## Abstract

**Purpose:**

The aim of our study was to determine whether delta red blood cell distribution (ΔRDW) improves neurological outcomes in acute ischemic stroke (AIS) patients 2 years after intravenous thrombolysis (IVT) therapy.

**Methods:**

AIS patients who received IVT between January 2013 and December 2019 were retrospectively analyzed. In accordance with their mRS scores, the patients were divided into two groups. A binary logistic regression analysis was conducted to determine the influencing factors of adverse functional outcomes. It was decided to evaluate the variables' the predictive ability by using the area under the receiver operating characteristic. For the poor neurological recovery risk model, features were selected using the LASSO regression model. We also developed a predictive model based on logistic regression analysis, which combined the features selected in the minimum absolute contraction and selection operator regression models. An evaluation of the discrimination, calibration, and clinical applicability of the predictive model was conducted using the C index, calibration chart, and decision curve analysis. Internal validation was evaluated via bootstrapping.

**Results:**

Binary logistic regression analysis showed that ΔRDW was an independent influencing factor for poor neurofunctional outcomes. The most appropriate ΔRDW cut-off value for predicting the recovery of poor neurological outcomes was 18.9% (sensitivity: 89.9%, specificity: 78.6%, *p* < 0.001). The predictive factors included in the nomogram were age, the occurrence of CHD, stroke, AF, ΔRDW, NIHSS score at onset, interval time from onset to IVT, and whether there were indwelling urine catheters and gastric tubes. The model has not only a good discrimination ability, which was indicated by an overall C index of 0.891 (95% confidence interval: 0.829–0.953), but also a considerable calibration ability. Decision curve analysis showed that the nomogram of adverse neurological outcomes recovery was useful in the clinical practice when intervention was implemented above the threshold of 1% possibility of adverse neurological outcomes recovery.

**Conclusion:**

In patients with AIS after thrombolysis, the ΔRDW is a potential influencing factor that can be readily used to predict the likelihood of poor neurological function recovery.

## Introduction

Acute ischemic stroke (AIS) is a condition caused by blood circulation disorders and ischemia-hypoxia of the brain that causes tissue necrosis. One of the most common forms of stroke is an ischemic stroke, which is characterized by a focal brain infarction, along with sudden and persistent neurological deficits. Globally, a stroke, whether ischemic or hemorrhagic, is the most common cause of serious adverse events, like deaths and disability (1, 2). AIS is a severe acute disease of the nervous system that accounts for over 65% of all stroke cases (3). Intravenous thrombolysis rapidly restores blood flow (perfusion), which is vital for neuronal survival and recovery, and improves clinical outcomes. However, it is estimated that only 2–5% of stroke patients have been treated with intravenous recombinant tissue plasminogen activator (rt-PA) (4), although patients suffering from AIS responded well to intravenous rt-PA treatment (5). Furthermore, the effective therapeutic time window for cerebral reperfusion via thrombolysis is limited to approximately 4.5 h after ischemia (6, 7). Due to its complications of ischemic stroke, which worsened patients' prognosis, treatment with rt-PA should be carefully considered, both for risks and benefits. Hence, better decision strategies and effective implementation are of urgent need to mitigate the disease burden of ischemic stroke in China (8).

Red blood cell distribution width (RDW) could be obtained from a complete blood count (CBC), which is a convenient and inexpensive way to measure erythrocyte size variability. Anemia and inflammation are commonly assessed by RDW clinically. The normal range of RDW is 11.0 to 16.0%, and it can rise under some pathological or even physiological conditions (9, 10). Anemia could be diagnosed, classified, and treated guided by analyzing the size of erythrocytes using RDW. Previously, Higher RDW used to be closely related to increased mortality in patients with cardiovascular and cerebrovascular events, such as ischemic stroke, coronary disease, and peripheral artery disease (11–13). Nowadays, clinical diagnosis of AIS relies on history, neurological examinations and neuroimaging, while several scoring systems and RDW are adopted to assess the severity of stroke (14). There is still no surrogate biomarker to diagnose stroke. RDW by flow cytometry, on the other hand, is one of the most promising optional markers for predicting the occurrence of stroke and prognosis after rt-PA therapy in ischemic stroke.

Studies showed that RDW change from day 1 to day 4 was an independent predictor of mortality in patients with community-acquired pneumonia (15). However, there is no literature reporting the association between the difference in RDW from before to after thrombolysis (ΔRDW) and AIS, especially neurological recovery after rt-PA therapy. It is hypothesized that changes in ΔRDW may affect neurological outcome in acute stroke patients. In this study, we sought to evaluate the prognostic effect of RDW difference on mRS score in patients with AIS 2 years after rt-PA therapy, which may be. It is of great significance for the prognostic analysis and treatment of acute ischemic cerebral infarction.

## Subjects, materials and methods

### Subjects and designs

We got a formal ethical approval from the Shanghai East Hospital Ethics Committee prior to implementation. Clinical, laboratory, and neuroimaging data of 361 AIS patients who received reperfusion therapy with intravenous thrombolysis (IVT) consecutively were retrospectively analyzed at Shanghai East Hospital between January 2013 and December 2019. Stroke-specialized neurologist diagnosed AIS based on radiographic and clinical findings from a brain imaging study.

#### Criteria for inclusion and exclusion

Including criteria: (1) As defined by Guidelines for the Diagnosis and Treatment of AIS (16). (2) Rt-PA intravenous thrombolysis was performed based on the latest guidelines of the American Heart Association (ASA) (17). (3) All patients and family members signed informed consent forms.

#### Excluding criteria

(1) Patients with malignant tumors and severe heart, liver, or kidney dysfunction; (2) Patients with cerebral hemorrhage, imaging changes of early large-scale cerebral infarction by craniocerebral computed tomography (CT) examination or epilepsy; (3) Patient with contraindications to thrombolysis; (4) Patients that was treated with anticoagulant. (5) Patients under the age of 18, or with incomplete clinical or laboratory data.

Neurological deficits were graded from 0 to 42 using the NIH Stroke Scale (NIHSS) score (18) and a higher score indicates a more severe condition. In addition to collecting demographic information, neurologists trained and certified in neurology collected modified Rankin Scale (mRS) scores. We grouped the patients according to the mRS score system (19), which was used to measure the outcome of functional recovery after stroke by the following: 0–2 being favorable and 3–6 being unfavorable. We recorded the NIHSS and mRS scores of patients on admission, discharge, and 2 years after hospital discharge.

### Observation of indexes

We documented the baseline characteristics, including gender, age, cerebrovascular risk factors (hypertension, diabetes mellitus, heart disease, current smoking and drinking, blood glucose (BG) and blood lipid on admission. The ethylene-diamine-tetra-acetic acid (EDTA) tubes were used for collecting blood samples on admission and the third day after IVT, respectively. Hematology automated analyzer XE-2100 (Sysmex Company, Japan) was used to conduct the CBC within 20 min (min) after the sample was collected. ΔRDW values were calculated as the difference between the RDW value measured upon the third day after IVT and on admission (i.e., [the third day after IVT hospitalization RDW] - [on admission RDW]). All the enrolled subjects underwent CT imaging using an 64-detector row CT scanner (Philips brilliance, Japan) and MR imaging using a 3.0 T scanner (Discovery MR750, GE Healthcare, Milwaukee, WI, USA) on admission or in outpatient in this study.

### Follow-up and study endpoints

A 2-year follow-up was performed on all patients to track their neurological recovery. A favorable functional outcome was defined as a mRS score of 0–2 and an unfavorable functional outcome as 3–6 based on the mRS score system. 2-year mortality was the endpoint. Outpatient visits and telephone follow-ups were used to collect follow-up information.

### Statistical analysis

The patients' baseline characteristics were presented as the total percentage and the mean ± SD in the categorical variables or median and interquartile range (IQR) in the continuous variables based on the normality of the distribution. Pearson's χ test or Fisher's exact test Student's *t*-test and Mann-Whitney U test were selected to calculate the differences for numerical and categorical variables, as appropriate.

The least absolute shrinkage and selection operator (LASSO) regression technique was adopted for the selection of data dimension and predictors. Models incorporating multivariate logistic regression were used to determine risk levels and develop a nomogram for poor prognoses. The probability of each variate contributing to the outcomes was assigned based on the regression coefficient value for each variate in our study. To further assess the accuracy of the nomogram in predicting patient prognoses, plotted the receiver operating characteristic (ROC) curve and calculated the area under curve of ROC (AUC) for receiver operating characteristics. The calibration curve was then introduced to evaluate the consistency of prediction and observation. In order to evaluate the clinical applicability of the nomogram, the decision curve analysis (DCA) was conducted by quantifying the net benefit across all threshold probabilities. Internal validation completed by the bootstrapping method (Resampling = 1,000).

The statistical analysis in this article was performed using R software (version 4.2.0, https://www.r-project.org). The nomogram was generated using the “rms” package of R software. Statistical significance levels reported were all two-way, with *P* < 0.05 considered statistically significant.

## Results

### Patients' characteristics

A total of 361 patients visited our clinic from January 2013 to December 2019. All participants were presented in Table 1 with their baseline characteristics. A mean age of 66 years was found among the patients, including the 129 women (35.7%). The cohort consisted of 248 participants with hypertension, 56 with CHD, 80 with AF, and 42 with stroke (previous stroke history). One hundred and forty two patients (39.3%) had a history of tobacco smoking and alcohol consumption was mentioned in 86 patients (23.8%). The median ΔRDW, LDL, and BG concentrations were 0, 2.75, and 7.07 mmol/L. The mean interval time from stroke onset to IVT was 180.00 min. All patients were divided into good and poor neurological outcomes recovery groups based on mRS scores. Table 1 provided all the clinical data regarding demographics, disease features, and treatment in each two groups.

**Table 1:** Baseline characteristics.

	**Values**
No. of patients	361
Age (median [IQR]), years	66.00 [59.00, 75.00]
Gender, female, *n* (%)	129 (35.7)
Onset to thrombolysis (median [IQR]), minutes	180.00 [140.00, 220.00]
ΔRDW (median [IQR]), *n* (%)	0.00 [−0.20, 0.30]
LDL (median [IQR]), mmol/L	2.75 [2.22, 3.44]
BG (median [IQR]), mmol/L	7.07 [5.99, 8.87]
Hypertension, *n* (%)	248 (68.7)
DM, *n* (%)	98 (27.1)
Smoking, *n* (%)	142 (39.3)
Drinking, *n* (%)	86 (23.8)
CHD, *n* (%)	56 (15.5)
Stroke, *n* (%)	42 (11.6)
AF, *n* (%)	80 (22.2)
AF drug, *n* (%)	7 (1.9)
Intravascular thrombectomy, *n* (%)	25 (6.9)
**Cerebral infarction**	
Large-artery atherosclerosis, *n* (%)	121 (33.5)
Small-vessel disease, *n* (%)	178 (49.3)
Cardioembolic, *n* (%)	57 (15.8)
Other cause, *n* (%)	4 (1.1)
Unknown cause, *n* (%)	1 (0.3)
Hemorrhage transformation, *n* (%)	55 (15.2)
Urine tube placed, *n* (%)	78 (21.6)
Stomach tube placed, *n* (%)	81 (22.4)
NIHSS at onset (median [IQR])	4.00 [2.00, 10.00]
NIHSS at discharge (median [IQR])	3.00 [1.00, 9.00]

RDW, red blood cell distribution width; LDL, low density lipoprotein; CHD, coronary heart disease; BG, blood glucose; DM, diabetes mellitus; AF, atrial fibrillation; NIHSS, the national institutes of health stroke scale; ΔRDW is equal to [the third day after thrombolysis RDW -on admission RDW].

### Feature selection

**Figure 1:**
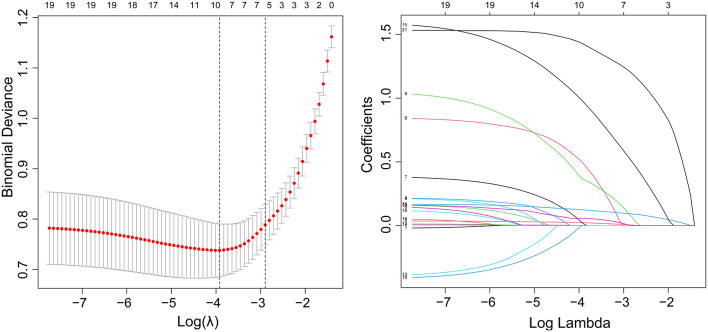
Demographic and clinical feature selection using the LASSO binary logistic regression model. **(A)** Fivefold cross-validation of the LASSO model's optimal parameter (lambda) was used. Plotted against log (lambda) is the partial likelihood deviance (binomial deviance). A minimum criteria and a 1 SE of the minimum criteria (the 1-SE criteria) were used to draw the dots representing the optimal values. **(B)** Profiles of the 21 features calculated by LASSO coefficients. This function plots coefficients vs. log (lambda) sequence. When lambda was optimal, there were nine features with non-zero coefficients, which was drawn as a vertical line. SE stands for standard error, and LASSO stands for least absolute shrinkage and selection operator.

Among all the demographics and clinical parameters, nine out of 21 features were thought to be potential predictors based on 361 patients in the cohort (Figures 1A,B) and showed non-zero coefficients in the LASSO regression model. These features included age, the occurrence of CHD, AF, stroke, ΔRDW, NIHSS score at onset, interval time from onset to IVT, and whether there were indwelling urine catheters and gastric tubes (Table 2).

**Table 2:** Univariate and multivariate analysis of risk factors for poor neurological outcomes recovery.

**Variables**	**Univariate**		**Multivariate**	
	**OR (95% CI)**	***p***	**OR (95% CI)**	***p***
Age	0.951 (0.929–0.973)	< 0.001	0.963 (0.932–0.994)	0.022
CHD	0.522 (0.288–0.947)	0.032	0.669 (0.281–1.593)	0.364
History of stroke	0.405 (0.21–0.783)	0.007	0.385 (0.154–0.964)	0.041
AF	0.247 (0.146–0.418)	< 0.001	0.616 (0.293–1.295)	0.201
NIHSS at onset	0.807 (0.768–0.848)	< 0.001	0.858 (0.805–0.916)	< 0.001
ΔRDW	0.153 (0.083–0.283)	< 0.001	0.212 (0.100–0.448)	< 0.001
Urine tube placed	0.084 (0.047–0.15)	< 0.001	1.017 (0.372–2.780)	0.973
Stomach tube placed	0.062 (0.034–0.112)	< 0.001	0.181 (0.071–0.46)	< 0.001
Interval time from onset to IVT	1 (0.996–1.005)	0.919	-	-

RDW, red blood cell distribution width; CHD, coronary heart disease; AF, atrial fibrillation; ΔRDW is equal to (the third day after IVT RDW – on admission RDW).

### Development of an individualized and applicable predictive model

**Figure 2:**
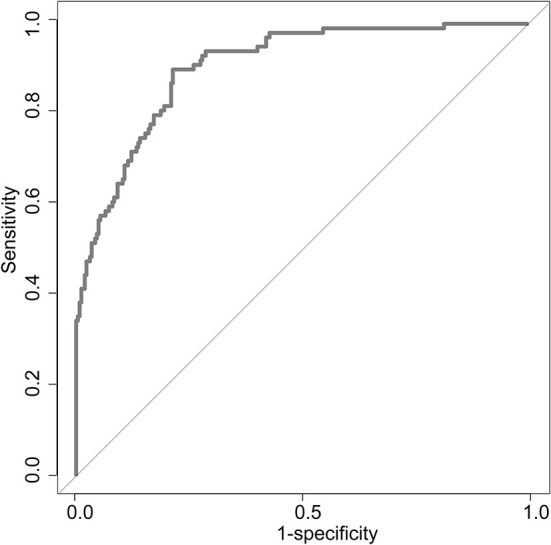
ROC curve to determine the predictive performance of ΔRDW for poor neurological outcomes recovery. ROC, Receiver operating characteristic; ΔRDW, the third day after IVT red blood cell distribution width – on admission red blood cell distribution width.

**Figure 3:**
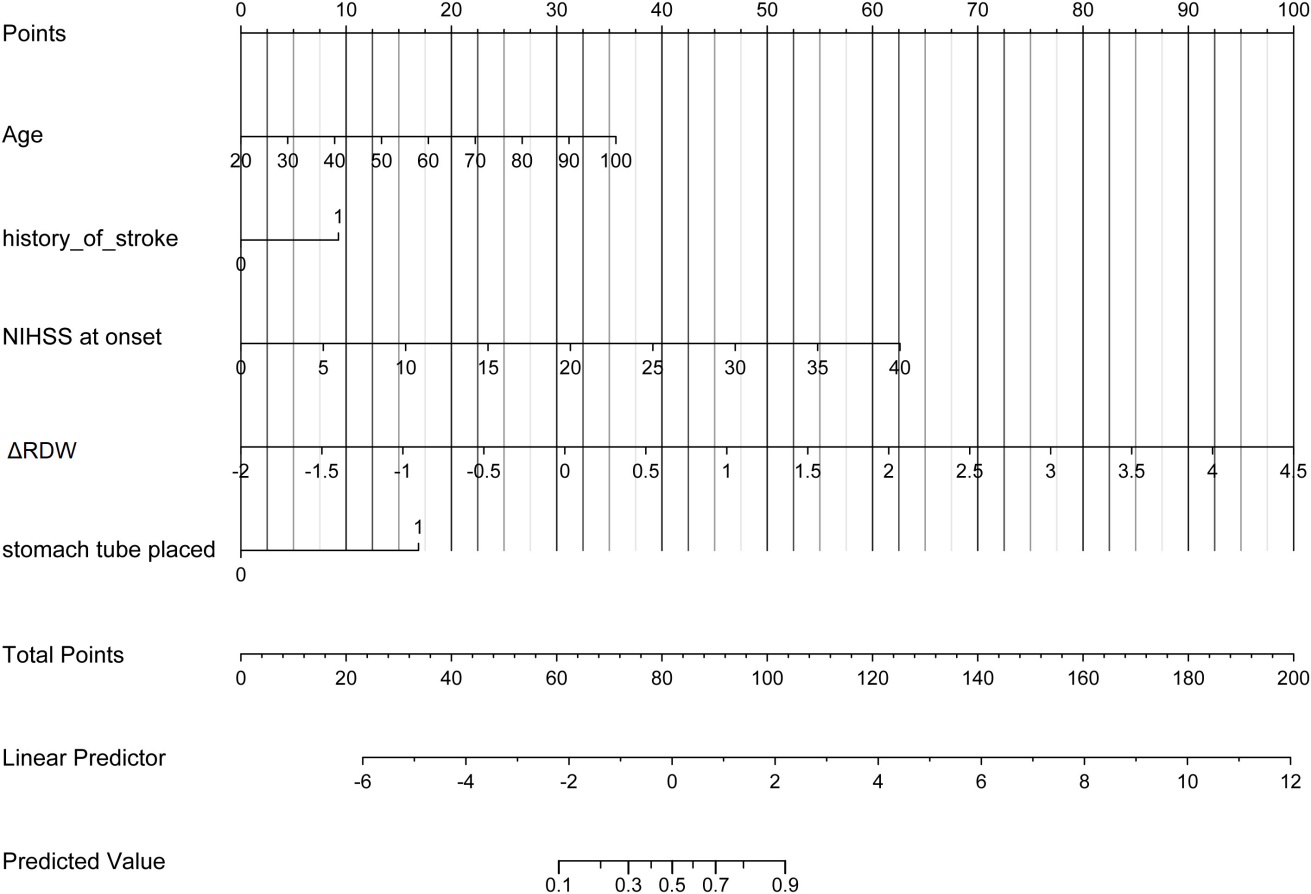
Developed poor neurological outcomes recovery nomogram. With the age, the history of stroke, NIHSS score at onset, ΔRDW, and indwelling gastric tubes in the cohort, a poor neurological outcomes recovery nomogram was developed. In our research, depending on the size of the regression coefficient, the nomogram scored the value of each variable. By converting each patient's score into a probability, the probability of different outcomes for each patient could be calculated. ΔRDW, the third day after IVT red blood cell distribution width – on admission red blood cell distribution width.

Uni-variable binary logistic regression analysis showed that each additional unit of age (*p* < 0.001), the incidence of CHD (*p* = 0.032), stroke (previous history of stroke) (0.007), and AF (*p* < 0.001), NIHSS score at onset (*p* < 0.001), ΔRDW (p < 0.001), and indwelling urine catheters (*p* < 0.001) and gastric tubes (*p* < 0.001) were significantly associated with patients' neurological outcomes recovery after thrombolysis for acute ischemic stroke (Table 2). AUC (ΔRDW) = 0.905 with a 95% confidence interval of AUC = 0.869 to 0.934 was obtained from and the statistically significant difference (*p* < 0.001) made by this model using ROC analysis (endpoint: poor neurological outcomes recovery) (see Figure 2). The optimal critical value for ΔRDW was 18.9%, while at this point with a sensitivity of 89.9% and specificity of 78.6% to calculated the maximum approximate index. Furthermore, in view of clinical parameters, multivariable binary logistic regression model demonstrated that every one unit increase in age (OR: 0.963; 95%CI: 0.932–0.994; *p* = 0.022), RDW (OR: 0.212; 95%CI: 0.100–0.448; *p* < 0.001), NIHSS score at onset (OR: 0.858; 95%CI: 0.805–0.916; *p* < 0.001), indwelling gastric tubes (OR: 0.181; 95%CI: 0.071–0.460; *p* < 0.001), and the occurrence of stroke (OR: 0.385; 95% CI: 0.154–0.964; *p* = 0.041) were considered to be independent predictors of patients' neurological outcomes recovery after stroke thrombolysis for AIS. An integrated model incorporating the aforementioned statistically independent predictors in multivariable binary logistic regression was designed and presented in the form of a nomogram in Figure 3.

### Apparent performance of the nonadherence risk nomogram and clinical application

**Figure 4:**
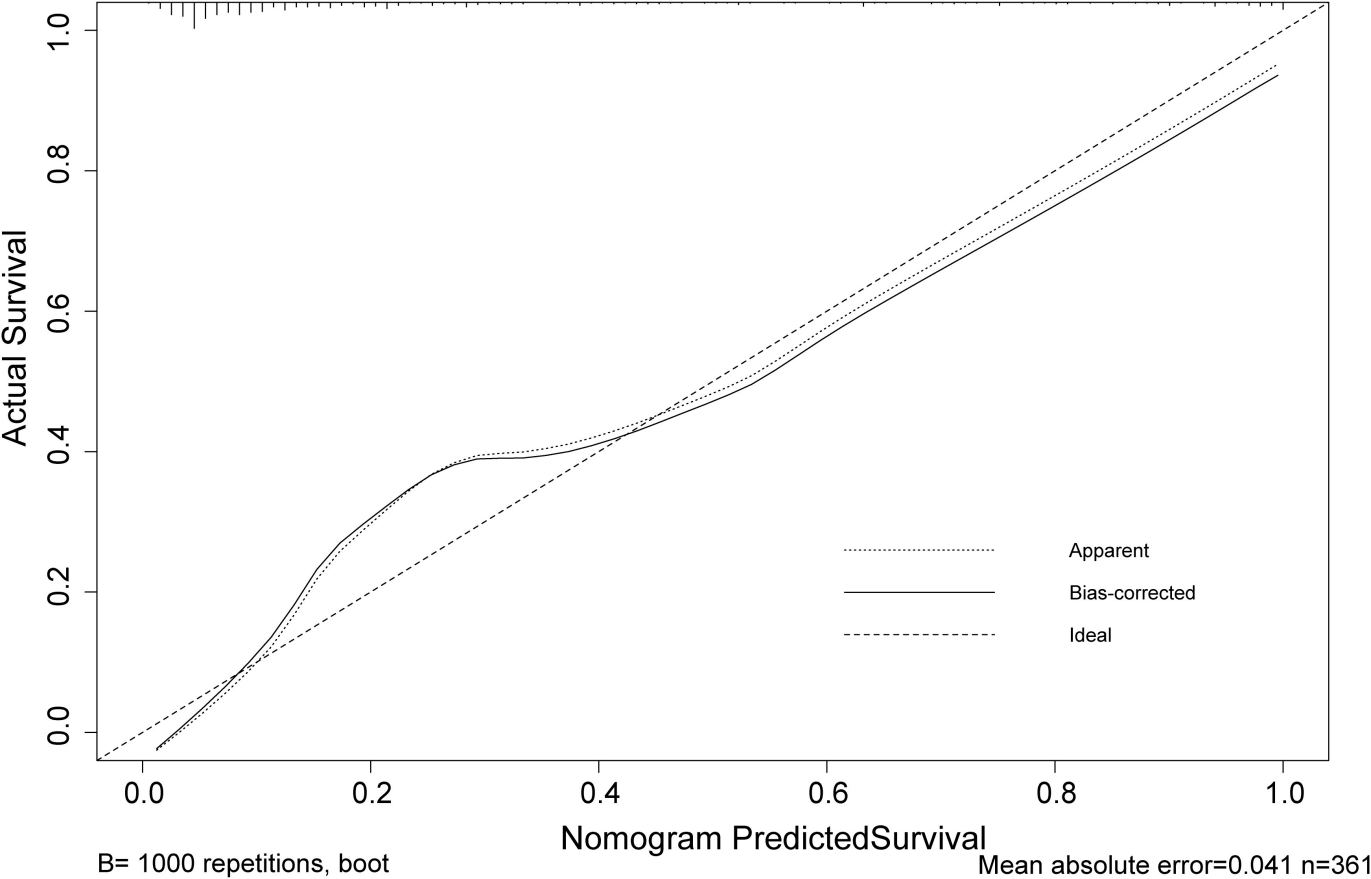
Calibration curve of the poor neurological outcomes recovery nomogram prediction in the cohort. Predicted poor neurological outcomes recovery risk is represented by the x-axis. The y-axis represents the actual diagnosed poor neurological outcomes recovery. Ideal model predictions are represented by the diagonal dotted line. The solid line represents the predictive performance of our nomogram, and a closer fit to the diagonal dotted line indicates a better prediction.

**Figure 5:**
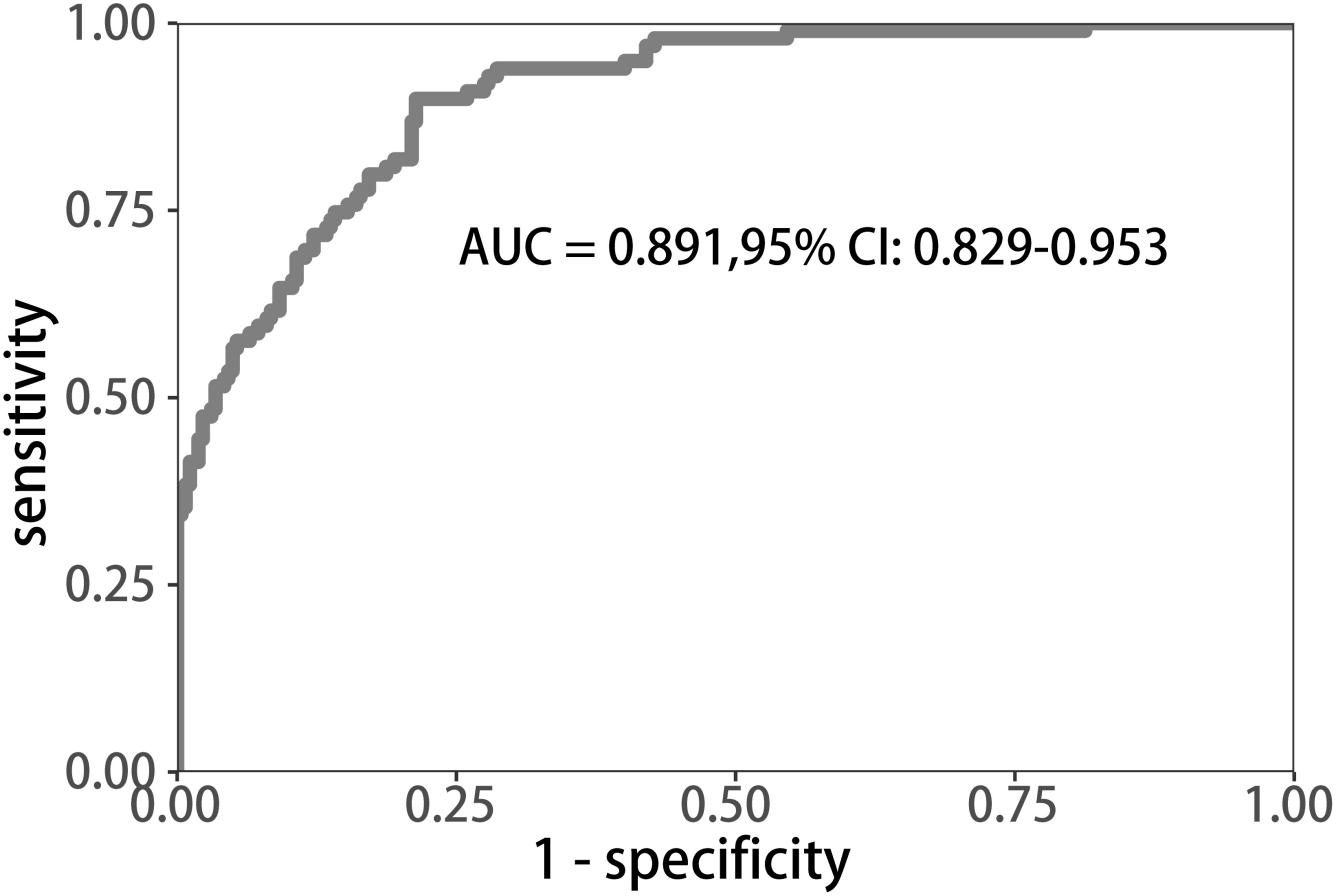
C-index analysis for the sensitivity and specificity of the nomogram.

**Figure 6:**
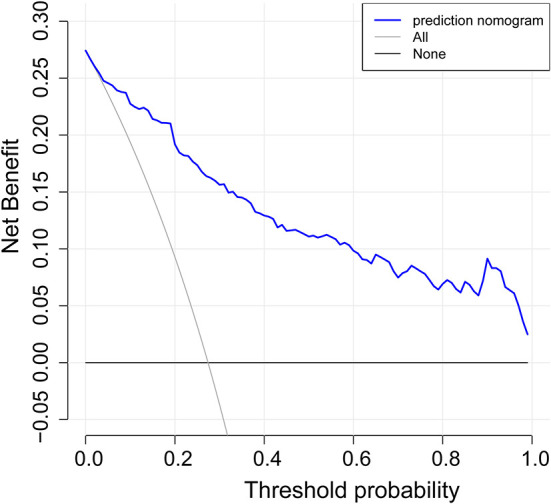
Decision curve analysis for the poor neurological outcomes recovery nomogram. The y-axis measures the net benefit. The blue line represents the poor neurological outcomes recovery risk nomogram. The light gray line represents the assumption that all patients suffer from poor neurological outcomes recovery. The black solid line represents the assumption that no patients suffer from poor neurological outcomes recovery.

The calibration curve of risk nomogram for predicting poor neurological outcomes recovery risk in patients after thrombolysis for AIS demonstrated a good fit (Figure 4). The C-index of the prediction nomogram was 0.891 (95% CI: 0.829–0.953) for the cohort and was confirmed through 1,000 bootstrapping validation, which suggested the model's good discrimination (Figure 5). The decision curve analysis for the poor neurological outcomes recovery nomogram was presented in Figure 6 and indicated that ΔRDW was a negative independent risk factor of poor neurological outcomes with clinical net benefit in a range of risk thresholds (threshold >1%) where the net benefit was comparable within this range, though there were several overlaps in the poor function recovery risk nomogram.

## Discussion

Table 1 and Supplementary materials showed that, compared to favorable functional outcome group after IVT, the unfavorable outcome group varied in not only ΔRDW, but also seven other features, including age, NIHSS score at onset or over, the occurrence of CHD, previous history of stroke, hemorrhagic transformation, incidence of thrombectomy and whether there were indwelling urine catheters or gastric tubes, which was in accordance with previous studies. Previous studies have shown that age identified as an unmodifiable risk factor for stroke (20), NIHSS score strongly affects stroke outcomes (21), hemorrhagic transformation after thrombolysis will affect prognosis (22), etc. It has been shown that thrombolytic therapy can improve the outcome of neurological injury and death in AIS patients when the indications for thrombolytic therapy are met, and contraindications are not present (16). In nursing and managing patients with AIS, proper and timely management plays an important role (23, 24), including an accurate diagnosis and prognosis. It is therefore necessary to determine the severity of neurological impairment and cognitive impairment on admission when treating AIS patients and to identify the risk factors that are associated with adverse outcomes following thrombolysis. Among many examinations, including physical examination and imaging examination, the value of routine laboratory parameters (such as RDW) is often underestimated. There has been an increase in the use of simple and inexpensive RDW tests in whole blood over the past few years to help predict many disease (25–27). As we all know, there are still a small portion of patients with cerebral infarction who suffer from serious conditions such as post-thrombotic hemorrhage after receiving thrombolytic therapy. Studies have shown that high RDW levels increase the risk of hemorrhagic transformation and stroke recurrence in patients with cerebral infarction receiving thrombolytic therapy (28, 29). Another research shows that high levels of RDW before thrombolysis can predict poor neurological recovery in patients with cerebral infarction at 1 year after IVT therapy (30) which means that RDW, as a fast, simple, and cheap method, is capable of distinguishing poor recovery of nerve function following thrombolysis. According to the ROC curve, the appropriate cutoff value for detection was 0.189, with 0.786 specificity and 0.899 sensitivity in our study. Furthermore, a logistic regression model was developed using several clinical and demographic parameters, including age, CHD, history of stroke, AF, ΔRDW, indwelling catheters, and the NIHSS score at the onset and after thrombolysis and was proven to have good discrimination and calibration capabilities. The results showed that the changes in RDW at the time of admission and that on the third day after thrombolysis were associated with poor prognoses for neurological outcomes. The decision curve analysis showed that when the intervention is implemented above the threshold of 1% of the possibility of poor neurological recovery, the nomogram of poor neurological recovery had a certain practical value in clinical practice. Previous studies have shown that high RDW levels are closely related to complications and death risks of various diseases (31–33). For example, RDW can be used as a prognostic factor for the severity and progression of AIS after antithrombotic therapy (34). Other studies have shown that RDW may become a biomarker of a high inflammatory response (35). Nevertheless, the biological mechanism that underlies the changes in RDW before and after thrombolysis and the recovery of function following thrombolysis in AIS remains unclear, inflammation and oxidative stress have been suggested as potential mechanisms.

The increase in RDW level can be attributed to the following reasons: (1) It is possible that metabolic pathway disorders contribute to the increase in RDW level (36, 37). (2) Since the increase in RDW levels is directly related to hypertension, a risk factor for cerebrovascular disease, a relationship between RDW and inflammation is highlighted, which supports the hypothesis that chronic inflammation contributes to elevated RDW levels (38). (3) As a result of large red blood cells occluding the carotid arteries, RDW may promote the progression of ischemic stroke (39). (4) An increase in RDW levels is associated with oxidative stress and the production of free radicals, which can further contribute to the occurrence of atherosclerosis following an ischemic stroke (40). (5) Studies have suggested that malnutrition may be a contributing factor to the increase in RDW level (41). (6) There is a correlation between an increase in RDW level and cerebrovascular diseases, suggesting that it may play a role in the changes in cerebral hemodynamics (42). Erythropoietin-induced erythrocyte maturation is inhibited by proinflammatory cytokines (43), which is partially reflected in an increase in RDW. Then, an inflammatory response to the body can affect bone marrow function and iron metabolism (44, 45). Moreover, oxidative stress has been implicated in increased RDW by shortened RBC survival and increasing the number of large premature RBC (46). Hence, resolution of inflammation and oxidative stress after thrombolysis in acute cerebral infarction may reduce RDW level.

It appears that cerebrovascular events may be a trigger for red blood cell abnormalities in AIS events as well as after thrombolysis. Likewise, regression analysis found a significant correlation between the changes in RDW before and after thrombolysis and the recovery of neurological function. Consequently, RDW represents a valuable diagnostic biomarker in these patients. In this regard, abnormal erythropoiesis and metabolic processes might lead to acute cerebrovascular disease or even exacerbate it. It is well-known that red blood cells, as oxygen carriers in the blood, can increase RDW level when their structures are abnormal, thus promoting thrombosis (47). Secondly, anisocytosis can cause a rise in nitric oxide, decreasing blood flow-dependent arterial dilatation, triggering ischemic injury events, or aggravating previously existing ischemic injuries.

It is true that this study has some limitations. First, patients' compliance varies after discharge. It is possible for some patients to die as a result of other major diseases or accidents. Additionally, we did not perform a stratified analysis of risk factors. Meanwhile, we only investigated the relationship between ΔRDW value and poor neurological outcomes. Considering that other factors may be influenced RDW level, continuous monitoring may be necessary. Finally, this clinical study focused more on correlation than causality, without exploring the mechanism behind the relationship. The conclusion needs to be supported and verified by further animal experiments. We will also conduct multicenter research in our next experiment to obtain more reliable results. Moreover, larger sample sizes and multicenter studies are required to evaluate our results, primarily to determine whether ΔRDW affected the results and if the results are clinically significant or not.

## Conclusion

Our study showed that ΔRDW is an independent risk factor for poor neurological recovery after thrombolysis in AIS patients. It is a reference value for treatment strategy for those patients with poor neurological recovery within 2 years of thrombolysis based on ΔRDW prediction.

## Data availability statement

The original contributions presented in the study are included in the article/supplementary material, further inquiries can be directed to the corresponding authors.

## Ethics statement

The studies involving human participants were reviewed and approved by the Shanghai East Hospital Ethics Committee. The patients/participants provided their written informed consent to participate in this study. Written informed consent was obtained from the individual(s) for the publication of any potentially identifiable images or data included in this article.

## Author contributions

Project administration, supervision, and resources: DH. Conceptualization, investigation, and resources: WY. Writing—review and editing: YW. Data curation and validation: AA. Methodology, funding acquisition, and visualization: CR. Formal analysis, writing—original draft, and software: YJ. All authors have read and agreed to the published version of the manuscript.

## Funding

This study was supported by the National Natural Science Foundation of China (Grant No. 81571277).

## Conflict of interest

The authors declare that the research was conducted in the absence of any commercial or financial relationships that could be construed as a potential conflict of interest.

## Publisher's note

All claims expressed in this article are solely those of the authors and do not necessarily represent those of their affiliated organizations, or those of the publisher, the editors and the reviewers. Any product that may be evaluated in this article, or claim that may be made by its manufacturer, is not guaranteed or endorsed by the publisher.

## Abbreviations

AIS: Acute ischemic stroke
rt-PA: recombinant tissue plasminogen activator
IVT: intravenous thrombolysis
CBC: complete blood count
ΔRDW: the third day after IVT RDW &#8211
on admission RDW
NIHSS: NIH Stroke Scale
mRS: Modified Rankin Scale
CT: computed tomography
3.0T MR: 3.0 Tesla magnetic resonance
EDTA: The ethylene-diamine-tetra-acetic acid
lasso: The least absolute shrinkage and selection operator
AUC: The area under the curve
BP: blood pressure
BG: blood glucose
CHD: coronary heart disease
AF: atrial fibrillation.
