# Supplementary material for: The difference in red blood cell distribution width from before to after thrombolysis as a prognostic factor in acute ischemic stroke patients: A 2-year follow-up
Source: Front Neurol. 2022 Oct 13;13:1011946. doi: 10.3389/fneur.2022.1011946 (PMC9606336; doi:10.3389/fneur.2022.1011946)
Supplement: Supplementary file 1 [file Data_Sheet_1.doc]

Patients’ characteristics

A total of 361 patients visited our clinic from January 2013 to December 2019. The baseline characteristics of study participants are shown in Table 1.

| Table 1. Baseline characteristics | | | | |
| --- | --- | --- | --- | --- |
|  | Total(n) | Favorable Functional Outcome （n=262） | Unfavorable Functional Outcome（n=99） | p-values |
| Females n(%) | 129（35.7） | 92（35.1） | 37（37.4） | 0.689 |
| age (median [IQR]) | 66.00 [59.00, 75.00] | 65.00 [58.00, 72.00] | 69.00 [64.00, 79.00] | 0.000 |
| Onset_to_thrombolysis (median [IQR]) | 180.00 [140.00, 220.00] | 185.00 [140.00, 220.00] | 180.00 [140.00, 215.00] | 0.918 |
| RDW3_1 (median [IQR]) | 0.00 [-0.20, 0.30] | -0.10 [-0.20, 0.20] | 0.30 [0.00, 0.80] | 0.000 |
| LDL (median [IQR]) | 2.75 [2.22, 3.44] | 2.75 [2.22, 3.51] | 2.80 [2.19, 3.27] | 0.238 |
| BG (median [IQR]) | 7.07 [5.99, 8.87] | 6.96 [5.91, 8.94] | 7.42 [6.1, 8.78] | 0.739 |
| History of hypertension，n (%) | 248 (68.7) | 176（67.2） | 72（72.7） | 0.310 |
| History of DM，n (%) | 98 (27.1) | 71（27.1） | 27（27.3） | 0.974 |
| smoking，n (%) | 142 (39.3) | 108（41.2） | 34（34.3） | 0.233 |
| drink，n (%) | 86 (23.8) | 66（25.2） | 20（20.2） | 0.321 |
| CHD，n (%) | 56 (15.5) | 34（13.0） | 22（22.2） | 0.030 |
| history_of_stroke ，n(%) | 42 (11.6) | 23（8.8） | 19（19.2） | 0.006 |
| AF，n (%) | 80 (22.2) | 39（14.9） | 41（41.4） | 0.000 |
| AF_drug_used ，n(%) | 7 ( 1.9) | 3（1.1） | 4（4） | 0.075 |
| Intravascular thrombectomy，n (%) | 25 ( 6.9) | 12（4.6） | 13（13.1） | 0.004 |
| Toast，n(%) |  |  |  | 0.507 |
| Large-artety atherosclerosis,n(%) | 121 (33.5) | 67（25.6） | 54（54.5） | - |
| Small-vessel disease,n(%) | 178 (49.3) | 172（65.6） | 6（6.1） | - |
| Cardioembolic,n(%) | 57 (15.8) | 19（7.3） | 38（38.4） | - |
| Other cause,n(%) | 4 ( 1.1) | 4（1.5） | 0（0） | - |
| Unknown cause,n(%) | 1 ( 0.3) | 0（0） | 1（1.0） | - |
| hemorrhage_transformation (%) | 55 (15.2) | 23（8.8） | 31（31.3） | 0.000 |
| urine_tube_placed (%) | 78 (21.6) | 24（9.2） | 54（54.5） | 0.000 |
| stomach_tube_placed (%) | 81 (22.4) | 22（8.4） | 59（59.6） | 0.000 |
| NIHSS_at_onset (median [IQR]) | 4.00 [2.00, 10.00] | 3.00 [2.00, 5.00] | 11.00 [6.00, 17.00] | 0.000 |
| NIHSS_at_over (median [IQR]) | 3.00 [1.00, 9.00] | 2.00 [0.00, 4.00] | 12.00 [6.00, 17.00] | 0.000 |
| RDW, red blood cell distribution width; LDL, low density lipoprotein; CHD, coronary heart disease; BG, blood glucose; DM, diabetes mellitus; AF, atrial fibrillation; NIHSS, the national institutes of health stroke scale; ΔRDW is equal to [the third day of hospitalization RDW - admission RDW]. | | | | |

Normality of the distribution：Shapiro-Wilk normality test

|  | W | p-value |
| --- | --- | --- |
| age | 0.98041 | 8.067e-05 |
| NIHSS_at_onset | 0.84572 | < 2.2e-16 |
| NIHSS_at_over | 0.81386 | < 2.2e-16 |
| Onset_to_thrombolysis | 0.99203 | 0.04999 |
| RDW3_1 | 0.73379 | < 2.2e-16 |
| LDL | 0.98429 | 0.0005764 |
| BG | 0.7485 | < 2.2e-16 |
